# Incidental thyroid carcinoma: Correlation between FNAB cytology and pathological examination in 1093 cases

**DOI:** 10.1016/j.clinsp.2022.100022

**Published:** 2022-03-18

**Authors:** Mariana Gonçalves Rodrigues, Luiz Fernando Ferraz da Silva, Vergilius José Furtado de Araujo-Filho, Letícia de Moraes Mosca, Vergilius José Furtado de Araujo-Neto, Luiz Paulo Kowalski, Paulo Campos Carneiro

**Affiliations:** a Departamento de Cirurgia, Cirurgia de Cabeça e Pescoço, Faculdade de Medicina FMUSP, Universidade de São Paulo, São Paulo, SP, Brazil; b Departamento de Patologia, Faculdade de Medicina FMUSP, Universidade de São Paulo, São Paulo, SP, Brazil

**Keywords:** Thyroid neoplasms, Incidental findings, Pathology, Diagnosis

## Abstract

**Objective:**

To investigate Incidental Thyroid Carcinoma (ITC) by comparing the results of Fine Needle Aspiration Biopsy (FNAB) cytology and the postoperative pathological findings.

**Methods:**

Data of 1479 patients who underwent total thyroidectomy were retrieved. Three hundred eighty-six patients were excluded due to insufficient data. Each surgical specimen studied received two histopathological diagnoses: the local diagnosis ‒ for the same area in which the FNAB was performed; and the final diagnosis, which includes a study of the entire surgical specimen.

**Results:**

A thousand and ninety-three patients were investigated. FNAB result was malignant in 187 patients, benign in 204, suspicious or indeterminate in 668 cases, and inconclusive in 34 cases. The prevalence of ITC was 15.1%. Most of the ITC in this series was less than 0.5 cm. The incidence of ITC was higher in Bethesda III (17.5% ITC) and IV (19% ITC) than in Bethesda II cases (1.5% false negatives and 9% ITC).

**Conclusion:**

Although the incidence of false-negative results in Bethesda II nodules is only 1.5%, 9% of these patients had ITC in the thyroid parenchyma outside the nodule that underwent preoperative FNAB. The incidence of ITC in the same scenario was even higher in Bethesda III (17.5%) and Bethesda IV cases (19%). Ultrasonography-guided FNAB is an excellent method for the assessment of thyroid nodules. However, biopsy sites should be carefully selected. Despite the high incidence of incidentalomas, total thyroidectomy should not always be the treatment of choice due to its rare but potentially serious complications. The findings of the present study can assist future clinical decisions towards active surveillance strategies for the management of papillary thyroid carcinoma.

## Highlights

• Incidental thyroid carcinomas are diagnosed by pathological study of the surgical specimen, with no previous suspicion by other diagnostic methods before surgery.
• Incidental thyroid cancer has been found at high rates in men and young people, usually, but not always, with more favorable histopathological characteristics and better prognosis.
• The better understanding of incidental thyroid cancer can improve decisions towards active surveillance strategies for the management of papillary thyroid carcinoma.

## Introduction

Thyroid cancer incidence has increased in the last years, and one of the main reasons for this fact is the rise in incidental detection of small nodules by imaging exams.^1^ Thyroid nodules are frequent in the population, especially in women and the elderly.^2^ They are identified by physical examination or incidentally detected in imaging tests requested for other reasons. In most cases, thyroid nodules are benign, but they are surgically treated when symptomatic or if malignancy is suspected.^3^ Fine-Needle Aspiration Biopsy (FNAB) guided by Ultrasonography (USG) is the most important investigation method and is essential for surgical treatment indication.^4^^,^^5^ The main purpose of investigating thyroid nodules is to diagnose malignancy.^2^ Thyroid carcinomas are called “incidental” when diagnosed by a pathological study of the surgical specimen, with no previous suspicion by other diagnostic methods before surgery. Incidental thyroid cancer rates have been evaluated by Smith et al.^6^ in a multicenter study that demonstrated high rates in men and young people. There are reports of patients with incidental thyroid carcinoma who did have an FNAB performed preoperatively, but those sampled only benign nodules, demonstrating the need for a more careful selection of the biopsy sites.^7^ When compared to non-incidental thyroid carcinomas, incidentalomas are more frequent in older patients. In earlier stages, they have more favorable histopathological characteristics and a better prognosis.^8^ Despite the more optimistic profile recorded in the literature, Maturo et al.^9^ identified evidence of lymph node metastases in 57.7% of patients with incidental thyroid carcinomas. Moreover, 4% of the 282 incidentalomas in a study conducted by Christakis et al.^10^ were classified as intermediate risk according to the ATA (American Thyroid Association) guidelines.

The present study aims to analyze the incidence of incidental malignant lesions in patients who underwent total thyroidectomy by comparing the results of Fine Needle Aspiration Biopsy (FNAB) cytology and postoperative pathological findings.

## Patients and methods

The present study is derived from a survey carried out in 2009‒2013 at Hospital das Clínicas of Faculdade de Medicina da Universidade de São Paulo (HCFMUSP), and the first results were published in 2018 by Mosca et al.^11^

Medical records of 1,479 patients who underwent total thyroidectomy and pathological examination of the resected thyroid were reviewed. FNAB results of preoperatively biopsied lesions were compared with the pathological studies conducted after thyroidectomy. Each surgical specimen investigated received two independent histopathological diagnoses: LD (Local Diagnosis) ‒ for the same area or nodule in which the FNAB was performed; and FD (Final Diagnosis), which includes the investigation of the entire surgical specimen. Of the 1479 individuals that underwent thyroidectomy, 248 were excluded due to insufficient general data, and another 138 were excluded due to the impossibility of correlating the area submitted to FNAB and the final pathological diagnosis.^11^ The Bethesda system was used in the interpretation of the results of FNAB cytology. For analysis purposes, Bethesda's categories were grouped into “benign” (Bethesda II), “atypical or suspicious” (Bethesda III, IV and V), “malignant” (Bethesda VI) and “nondiagnostic or unsatisfactory” (Bethesda I). Surgical indications for patients with a nondiagnostic result of FNAB or benign diagnosis were compressive symptoms due to goiter, hyperthyroidism refractory to non-surgical treatments, substernal goiter, and high clinical or radiological suspicion of malignancy.

The present study was approved by HCFMUSP Institutional Review Board (Comissão de Ética para Análise de Projetos de Pesquisa ‒ CAPPesq).

## Results

One thousand and ninety-three patients were investigated. Of these, 969 (88.7%) were female. The 'patients' mean age was 52.7, ranging from 13 to 87 years. FNAB results were as follows: malignant in 187 patients (17.1%), benign in 204 cases (18.7%), suspicious or indeterminate in 668 cases (61.1%), and inconclusive in 34 cases (3.1%). The pathological result of the site where FNAB was performed was benign in 201 of the 204 cases classified as Bethesda II. Therefore, 1.5% of the cases (3 patients) were false-negative in the preoperative cytological analysis. Two (1.1%) out of 187 patients classified as Bethesda VI were found to be false positive, as they had benign LD (Table 1). Of the 1,093 patients that underwent total thyroidectomy, 294 were diagnosed with malignancy at the biopsy site (LD). In the remaining 799 patients, LD was benign. Of these, 121 cases had a diagnosis of malignancy considering the histopathological evaluation of the entire surgical specimen (FD), characterizing, therefore, a 15.1% prevalence of incidental thyroid carcinoma.

**Table 1:** Association of preoperative FNAB Bethesda classification and local diagnosis at the final pathological report.

**Postoperative local diagnosis**	**Preoperative Bethesda findings**						
	**I**	**II**	**III**	**IV**	**V**	**VI**	**Total**
Adenoma	2	5	25	26	2	0	60
Goiter/Thyroiditis/Graves	30	196	314	189	9	2	740
Anaplastic carcinoma	0	0	0	1	0	1	2
Follicular carcinoma	0	0	3	8	3	0	14
Medullary carcinoma	1	1	0	0	0	6	8
Papillary carcinoma	1	2	41	16	28	171	259
Poorly differentiated carcinoma	0	0	1	2	0	0	3
Other types of carcinomas	0	0	0	0	0	6	6
Linfoma	0	0	0	0	0	1	1
**Total**	**34**	**204**	**411**	**242**	**42**	**187**	**1,093**

**Fig. 1:**
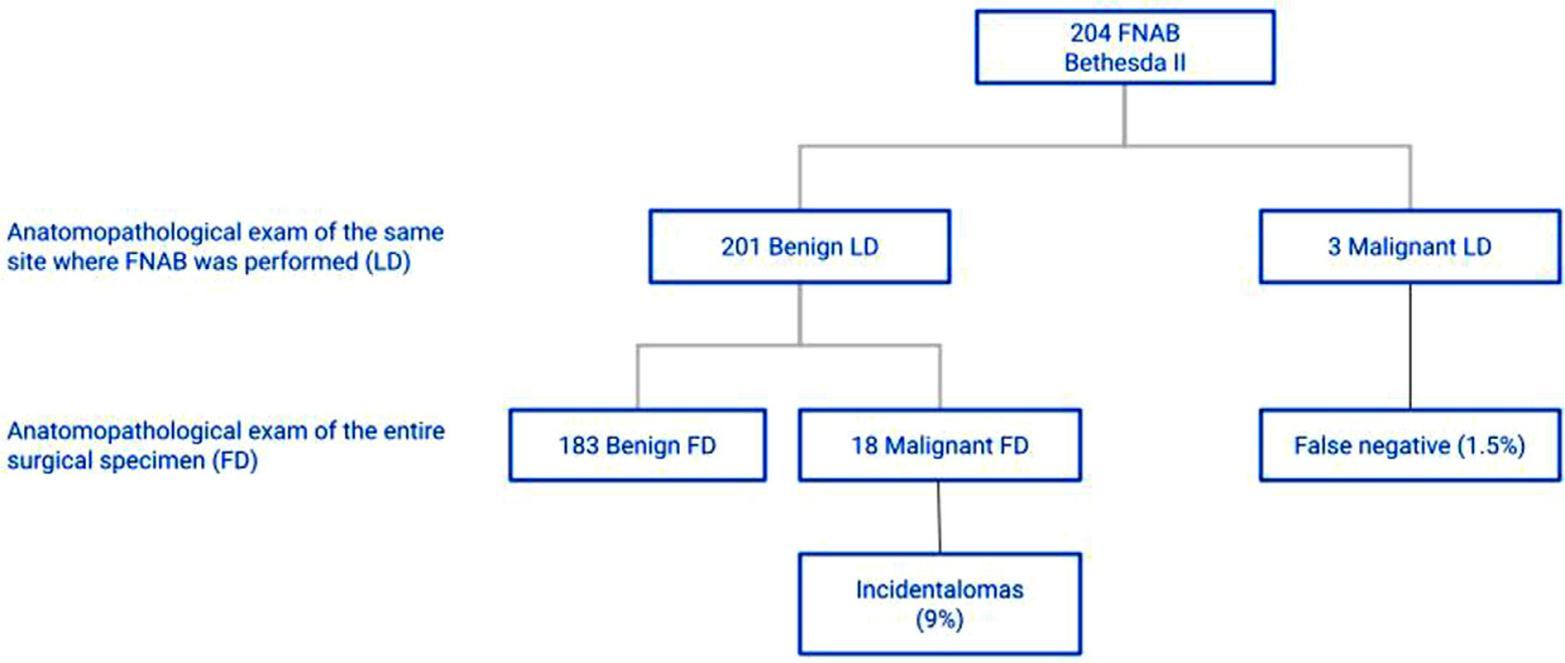
Anatomopathological exams of patients categorized as Bethesda II.

**Fig. 2:**
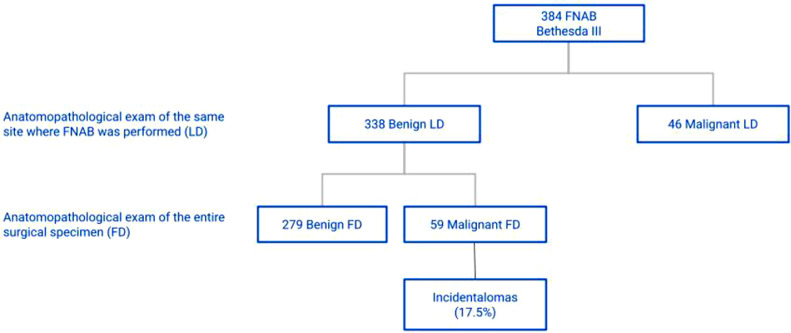
Anatomopathological exams of patients categorized as Bethesda III.

**Fig. 3:**
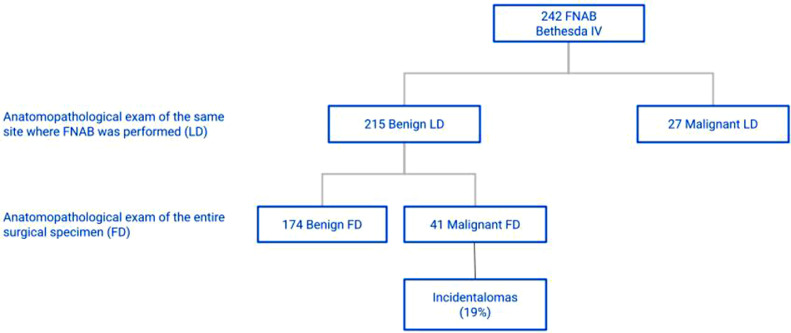
Anatomopathological exams of patients categorized as Bethesda IV.

**Fig. 4:**
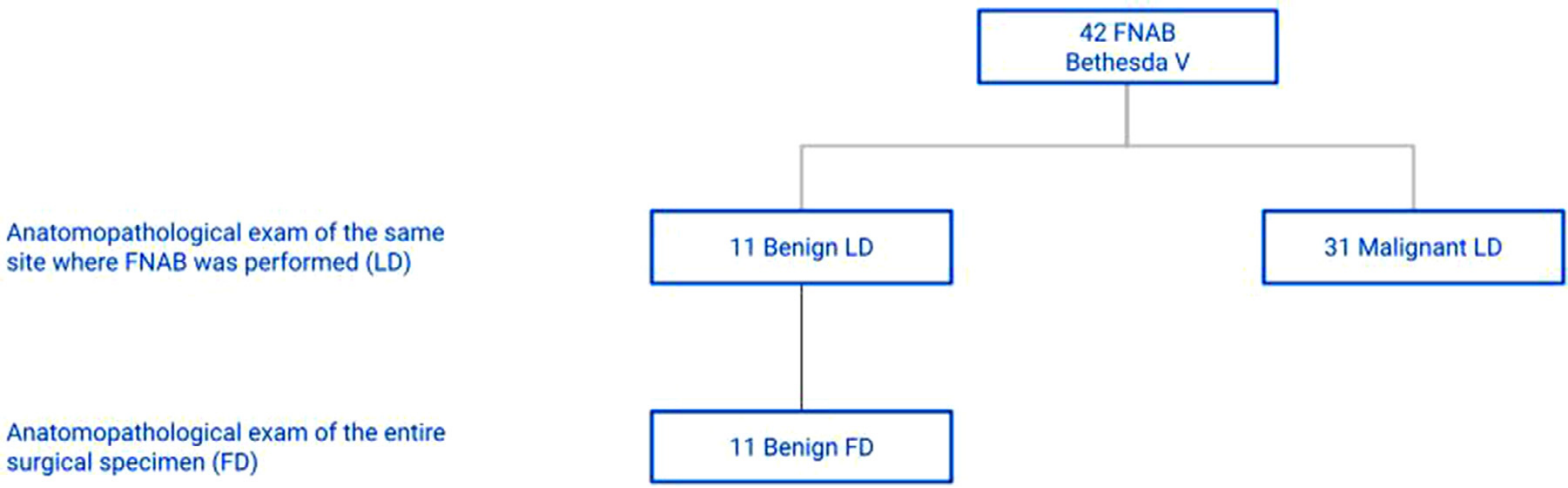
Anatomopathological exams of patients categorized as Bethesda V.

**Fig. 5:**
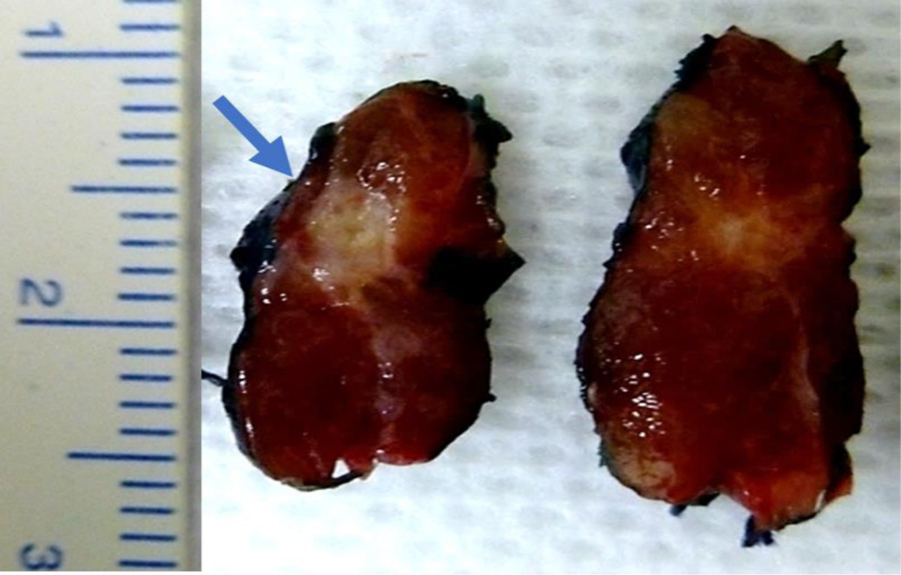
Gross appearance of an incidental papillary carcinoma with a major axis of 0.5 cm (blue arrow).

Considering the 201 patients classified as Bethesda II in FNAB and with benign LD, it was found that 183 had benign FD and 18 had malignant FD, characterizing a 9.0% prevalence of incidental diagnosis of malignancy in the group of patients categorized as Bethesda II (Fig. 1). The incidence of incidentalomas was higher in Bethesda III and IV groups (Fig. 2, Fig. 3). There was no incidental thyroid carcinoma in cases preoperatively diagnosed as Bethesda V (Fig. 4). Regarding the 'incidentalomas' size, 82 (67.8%) were smaller than or equal to 0.5 cm, 16 (13.2%) were between 0.5 and 1.0 cm (Fig. 5), and 23 (19%) were larger than 1cm on the major axis (Table 2). 'Incidentalomas' predominant histological type was papillary thyroid carcinoma (118 cases, 97.5%), but occurrences of follicular carcinoma, medullary carcinoma, and a carcinoma without other specifications were also found (Table 3).

**Table 2:** Size of incidentalomas.

**Tumor size**	**Number of incidentalomas (%)**
≤ 0.5 cm	82 (67.8%)
> 0.5 cm to ≤ 1.0 cm	16 (13.2%)
> 1.0 cm	23 (19.0%)

**Table 3:** Incidentalomas histological types.

Histological types	**Number of cases (%)**
Papillary carcinoma	118 (97.5)
Follicular carcinoma	1 (0.8)
Medullary carcinoma	1 (0.8)
Other types of carcinomas	1 (0.8)

## Discussion

The present study compared the result of preoperative cytological analysis of thyroid lesions with the respective pathological examination of the thyroidectomy surgical specimen. Pre and postoperative results were compared for determining malignancy or benignity. Preoperatively, the Bethesda system was used to determine the risk of malignancy of a biopsied nodule. Lesions classified as Bethesda II were considered benign, Bethesda VI as malignant and Bethesda III, IV and V as atypical or suspicious. Postoperatively, two different pathological evaluations were performed, which adds value to the interpretation of the results presented. Firstly, it was verified whether the nodule or the lesion biopsied by FNAB had a pathological diagnosis compatible with its preoperative cytological classification, that is if the diagnosis was confirmed. Only 3 (1.5%) of the lesions considered benign by FNAB were malignant in the local pathological exam (false-negative). The second review of the pathological exam verified whether there was malignancy in any region of the excised surgical specimen, and not only in the place where the biopsy was performed preoperatively. This second evaluation revealed the occurrence of incidental cancer in 15.1% of the patients with benign anatomical pathology at the site of the FNAB. Considering only the cases that were benign in FNAB (Bethesda II), the incidentaloma prevalence dropped to 9.0%. A multicenter study,^6^ published in 2013 by Smith et al., showed that incidental thyroid carcinoma rate reached 15.6% of the 1,523 patients operated for benign preoperative pathologies (excluding pathologies of undetermined significance and suspected for malignancy). Despite presenting similar incidentaloma rates, it is important to note that, while the first study compared the FNAB results with the final pathological examination, the present study compares the pathological examination result of the site in which FNAB was performed with the final pathological examination of the entire surgical specimen.

Corroborating the present study, Bradley et al.^12^ concluded that 12% of patients were operated on for benign conditions, such as Hashimoto's thyroiditis, follicular adenoma, multinodular goiter, and Graves' disease, had an Incidental Papillary Carcinoma (IPC) as their final histopathological diagnosis. The author even suggests that total thyroidectomy should be considered in patients with Hashimoto thyroiditis and follicular adenoma due to the high incidence of IPC in the contralateral lobe. A preference for total thyroidectomy as the first surgical treatment for benign diseases is also stated in other studies^6^^,^^9^^,^^12^, ^13^, ^14^, ^15^, ^16^ due to the high prevalence of incidental neoplasms. However, the authors recommend cautiousness and attention to the classic indications for total thyroidectomy in order to avoid rare but potentially serious complications, such as definitive hypoparathyroidism and inferior laryngeal nerve injury in patients without malignancy suspicion. When non-surgical intervention is indicated for patients with benign FNAB, the possibility of coexisting microcarcinoma with its variable prognosis should be taken into account and explained to the patient.^17^

Machala et al., in a study that also compared FNAB and thyroid anatomopathological results, concluded that the cytological method is essential for diagnostic investigation of thyroid lesions because it is safe, simple, fast, and cost-effective since it receives adequate pathological interpretation.^18^ New investigation methods of thyroid nodules, such as elastography, PET/CT, biomarkers, and tumor markers are being investigated, but there is a consensus that USG-guided FNA is essential for the management of patients with thyroid nodules.^3^, ^4^, ^5^^,^^8^ Nevertheless, the high occurrence of incidentalomas draws attention to the need for a more careful selection of thyroid sites to be biopsied. When only the LD results are compared with the preoperative diagnosis, it can be seen that the false-negative percentage in the Bethesda II category is 1.5% in this study, compatible with the error rate predicted for the method in the literature 0% to 3%.^19^ Thus, it can be affirmed that the high rates of incidental thyroid carcinoma are due to a thyroid biopsy site selection problem rather than failure of the FNAB cytological interpretation itself. Miccoli et al. reported that in some cases of incidental thyroid carcinoma, FNAB was performed preoperatively in another nodule, which was benign. The authors concluded that there is a need not only to select the biopsy sites more carefully but also to collect several samples from the same nodule when it exceeds 2 cm in diameter.^7^

Pezzolla et al.^20^ compared the occurrence of incidentalomas in patients undergoing thyroidectomy for presumably benign diseases and patients with Bethesda III nodules undergoing thyroidectomy. This study obtained 16.8% of incidentalomas in the first group and 16.2% of malignant lesions in the second, but not in the same biopsied site preoperatively diagnosed as Bethesda III. Attention should be given to the indications for FNAB in thyroid nodules: preferably nodules larger than 10 mm, solid, hypoechoic, taller-than-wide, with irregular margins, taller than wide, with extrathyroid extension, intranodular vascular spots, or microcalcifications.^18^^,^^21^^,^^22^ However, it may be necessary to optimize such indications in order to reduce not only the occurrence of postoperative diagnosis of malignant lesions but also the overdiagnosis of clinically insignificant thyroid neoplasms. In the present study, 19% of patients with an incidental malignant lesion had tumors larger than 1.0 cm, which, therefore, would be probably identifiable by preoperative USG. Active Surveillance (AS) is a clinical practice proposed for the management of selected Papillary Thyroid Microcarcinomas (mPTC) instead of the primary surgical approach. AS could be an alternative to immediate surgery to avoid overtreatment in unifocal intrathyroidal mPTC, without metastatic lymph nodes or aggressive cytological features. However, multifocality is a potential contraindication for this practice, as well as the subcapsular location of the mPTC, previous external radiotherapy, or family history of differentiated thyroid carcinoma.^23^ Although the authors of the present study believe that AS plays a key role in mPTC, the authors stress that the high incidence of incidental thyroid carcinoma should be considered when multifocality is suspected, which is a potential contraindication for AS, as mentioned above. Thyroid carcinoma prevalence identified at autopsies is high, reaching 35.6% of consecutive autopsied patients in a Finnish study,^24^ which concluded that papillary tumors smaller than 0.5 cm could be a frequent finding in the thyroid gland. In contrast, Christakis et al.^10^ published the occurrence of intermediate-risk incidentalomas (according to ATA 2015) in 4% of the incidental thyroid carcinomas in their series (282 incidentalomas/1,369 patients), highlighting the oncological relevance of such findings.

The present study also emphasizes the importance of performing anatomopathological analysis on the entire extension of the resected thyroid gland, and not only in the site of the lesion in focus, given the risk of incidental neoplasms.

## Conclusion

The ITC incidence is high. Although the occurrence of false-negative results in Bethesda II nodules is only 1.5%, 9% of these patients presented ITC in the thyroid parenchyma outside the nodule evaluated by preoperative FNAB. The incidence of ITC in the same scenario was even higher in Bethesda III (17.5%) and Bethesda IV cases (19%). Despite the high incidence of incidentalomas, the authors recommend cautiousness and attention to the classic indications for total thyroidectomy in order to avoid potentially serious complications. Most incidentalomas are less than 0.5 cm in the largest diameter. Papillary thyroid carcinoma is the predominant histological type. Anatomopathological examination of the surgical specimen should be performed in the entire specimen, and not only in macroscopic lesions. Ultrasonography-guided FNAB is an excellent method for thyroid nodules clinical evaluation. Nevertheless, the areas to be biopsied must be carefully selected.

## Authors' contributions

All authors contributed to this study. Data collection and case review were performed by Mosca L and Araujo-Neto VJ. Data analysis was supervised by Silva LF and Kowalski LP. Araujo-Filho VJ and Carneiro PC were responsible for the study design and data analysis The manuscript draft and its final version were written by Rodrigues MG. The final version of the manuscript was reviewed by  Silva LF, Kowalski LP, Araujo-Filho VJ and Carneiro PC. All approved the final manuscript. The corresponding author is Carneiro PC who attests that all participants in this study meet the criteria for authorship.

## Conflicts of interest

The authors declare no conflicts of interest.
